# Supplementary material for: Assessing microbiome population dynamics using wild-type isogenic standardized hybrid (WISH)-tags
Source: Nat Microbiol. 2024 Mar 19;9(4):1103–16. doi: 10.1038/s41564-024-01634-9 (PMC10994841; doi:10.1038/s41564-024-01634-9)
Supplement: Supplementary file 1 — Supplementary Figs. 1–14. [file 41564_2024_1634_MOESM1_ESM.pdf]

nature microbiology

Resource

https://doi.org/10.1038/s41564-024-01634-9

Assessing microbiome population dynamics using wild-type isogenic standardized hybrid (WISH)-tags

In the format provided by the authors and unedited

Supplementary Figures 1-14

Assessing microbiome population dynamics using wild-type isogenic standardized hybrid (WISH)-tags

Benjamin B. J. Daniel[1], Yves Steiger[1], Anna Sintsova[1], Christopher M. Field[1], Bidong D. Nguyen[1], Christopher Schubert[1], Yassine Cherrak[1], Shinichi Sunagawa[1], Wolf-Dietrich Hardt[1], Julia A. Vorholt[1]✉

[1]Institute of Microbiology, ETH Zurich, Zurich, Switzerland

Email: jvorholt@ethz.ch

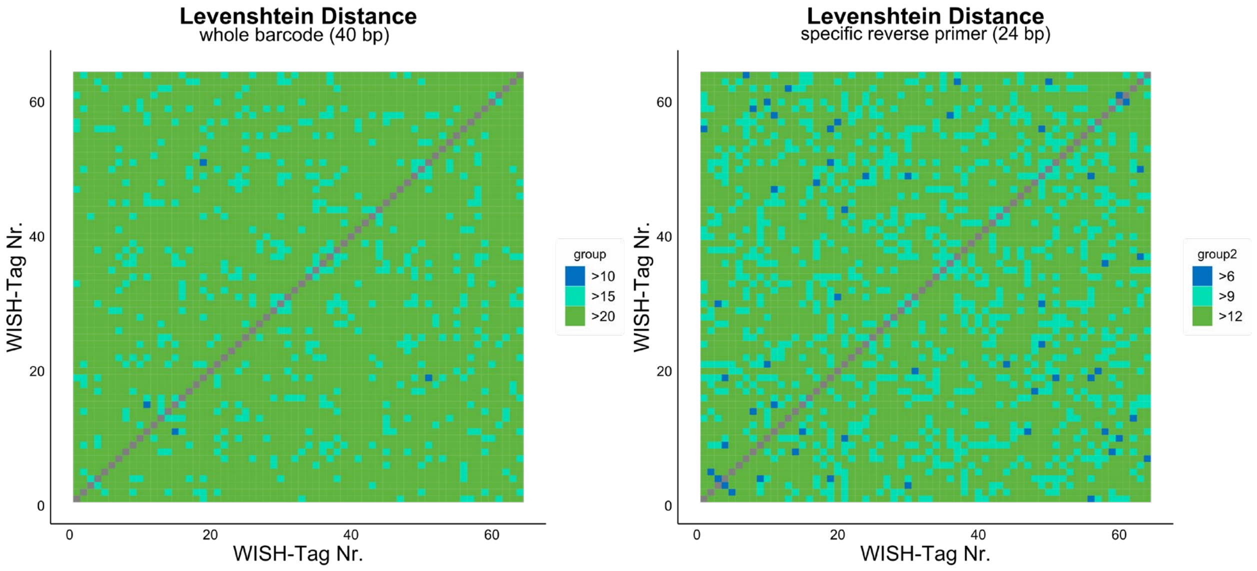

Supplementary Figure 1. The Levenshtein distances between the first 64 WISH-tags. This illustrates the minimal number of base exchanges required in one barcode to turn it into another, of WISH-tag sequences are compared, with the WISH-tags being ordered in increasing WISH-tag Number on the x and y axis, with the diagonal being greyed out to indicate self-comparison. Heatmap for the Levenshtein-distances between the barcodes and specific primers for the first 64 WISH-tags. The tresholds for the color code was adjusted between the entire barcode sequence (left) and the unique primer sequence (right) to reflect the total length of the tested sequences (40 and 24 bp, respectively).

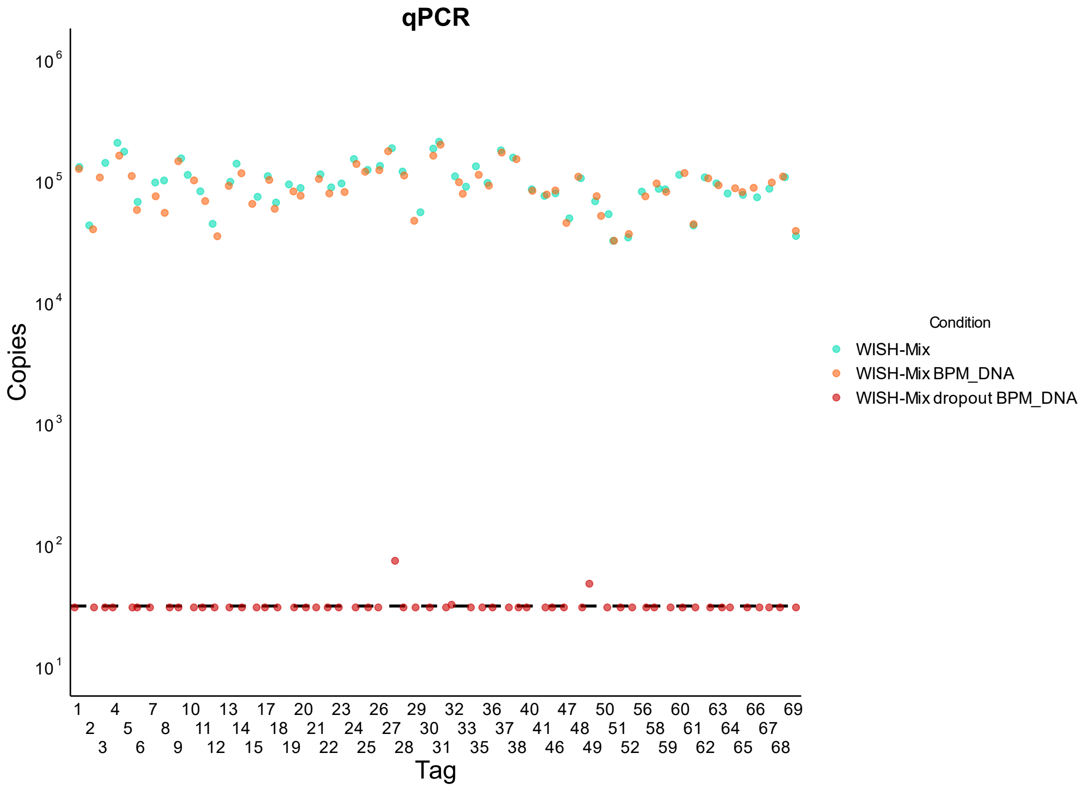

Supplementary Figure 2. Validation of WISH-Tags by qPCR (break-down of Figure 2a, right column). The x-axis shows each individual WISH-tag and the y-axis the copy number inferred by qPCR. The dashed line represents the detection limit as determined by the cutoff set at a cycle time of 31. Light blue points indicate the mix of all WISH-tags in the test, and orange points show the signal when 10 ng bacterial, plant and mouse DNA were added as well. The grey squares are the negative control, where the specific primer for the WISH-tag in question was used to amplify a mix of the same 10 ng bacterial, plant and mouse DNA in combination with a mix of all WISH-tags except for the one under investigation were used as template. BPM, bacteria plant mouse.

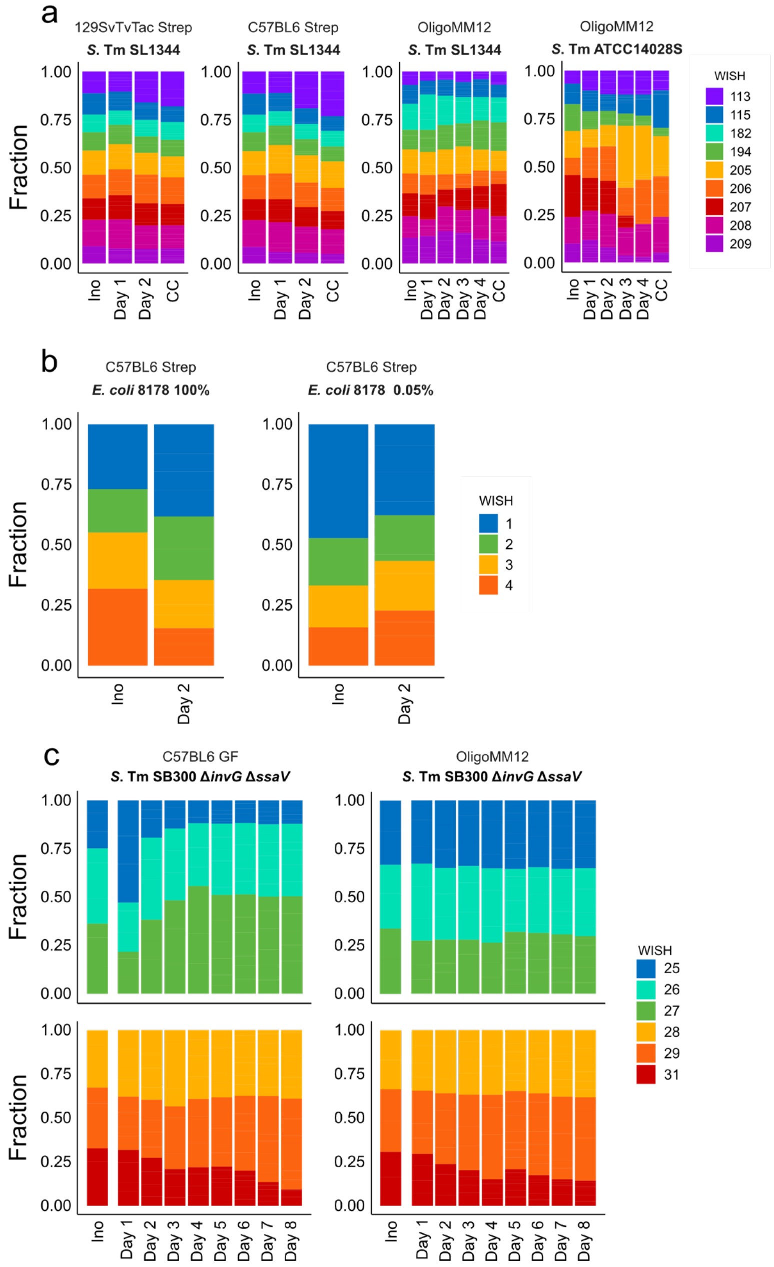

Supplementary Figure 3. Validation of barcoded lines for mouse gut associated strains. Fractional bar charts comparing the relative fitness of near isogenic lines created by integration of WISH-tags. The x-axis indicates the time the sample was collected. The y-axis indicates the fraction a line makes of the total population. The title above the individual plots conveys the mouse model used for the validation, and the bold text indicates the barcoded strain. a) Relative fitness between WISH-tagged *S.* Tm strains in different mouse backgrounds. The analysis of the DNA extracted from the mouse feces was analyzed using Illumina sequencing. The plot on the left shows the data for SL1344 barcoded with nine WISH-tags tested in 129S6/SvEvTac-mice (n=6). Plot two displays the data for SL1344 barcoded with nine WISH-tags tested in C57BL/6J-mice (n=6). Plot three shows the data for SL1344 barcoded with nine WISH-tags tested in the gnotobiotic C57BL/6 OligoMM12-mouse model that contains a microbial community of 12 strains (n=7)(ref.[5]). The plot on the right shows data for ATCC14028s barcoded with eight WISH-tags tested in OligoMM12-mice (n=7). b) No fitness defect is observed in the WISH-tagged murine commensal *E. coli* 8178 in streptomycin pretreated C57BL/6 mice. The experiment was performed with a population entirely comprised of barcoded *E. coli* 8178, as well as a population where the barcoded fraction was diluted to 0.05% using wild-type bacteria. In total, four different, near isogenic lines were tested (replicates: n=4). c) Comparison of the relative fitness of *S.* Tm barcoded near-isogenic lines for the avirulent SB300 Δ*invG* Δ*ssaV* [49]. The x-axis shows the day the samples were taken. The y-axis indicates the fraction of the total population. Each of the bars represents the averaged data for multiple mice (top left panel: n=6, top right panel: n=5, bottom left panel: n=7, bottom right panel: n=6), for the data resolved by mouse, see Supplementary Figure 6. The data was replotted from Figure 3bc. Ino, inoculum; CC cecum content.

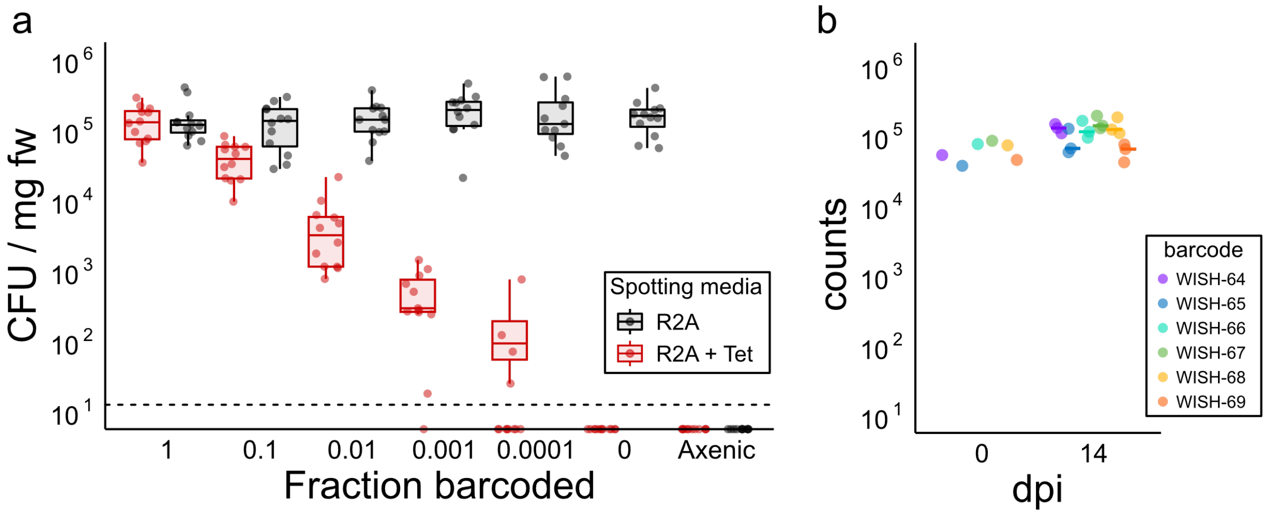

Supplementary Figure 4. *In planta* validation of tagged *Sphingomonas* Leaf257 to detect potential fitness effects due to tagging. Tagged *Sphingomonas* Leaf257 strains were diluted 10-fold against untagged strains (6 orders or magnitude). a) Absolute number of tagged and untagged strains. The x-axis indicates the share of the inoculum made up by the six barcoded lines. The y-axis indicates the CFU per mg plant fresh weight counted after plating the wash of from the plants at 14 dpi. The CFU for the black points were counted on non-selective media. The red points were counted on selective media, where only the barcoded fraction of the population could grow (n = 12 for all treatments). The center line represents the median, with the upper and lower edges of the box representing the 25 and 75 percentiles respectively. The whiskers extend at most 1.5 times the interquartile range. b) Quantification of WISH-tags in the inoculum and from the fully barcoded condition using Illumina sequencing after harvest and wash-off from the plant. The y-axis shows the counts retrieved per WISH-tag (n = 1 for 0 dpi and n = 3 at 14 dpi, the line represents the mean of all replicates).

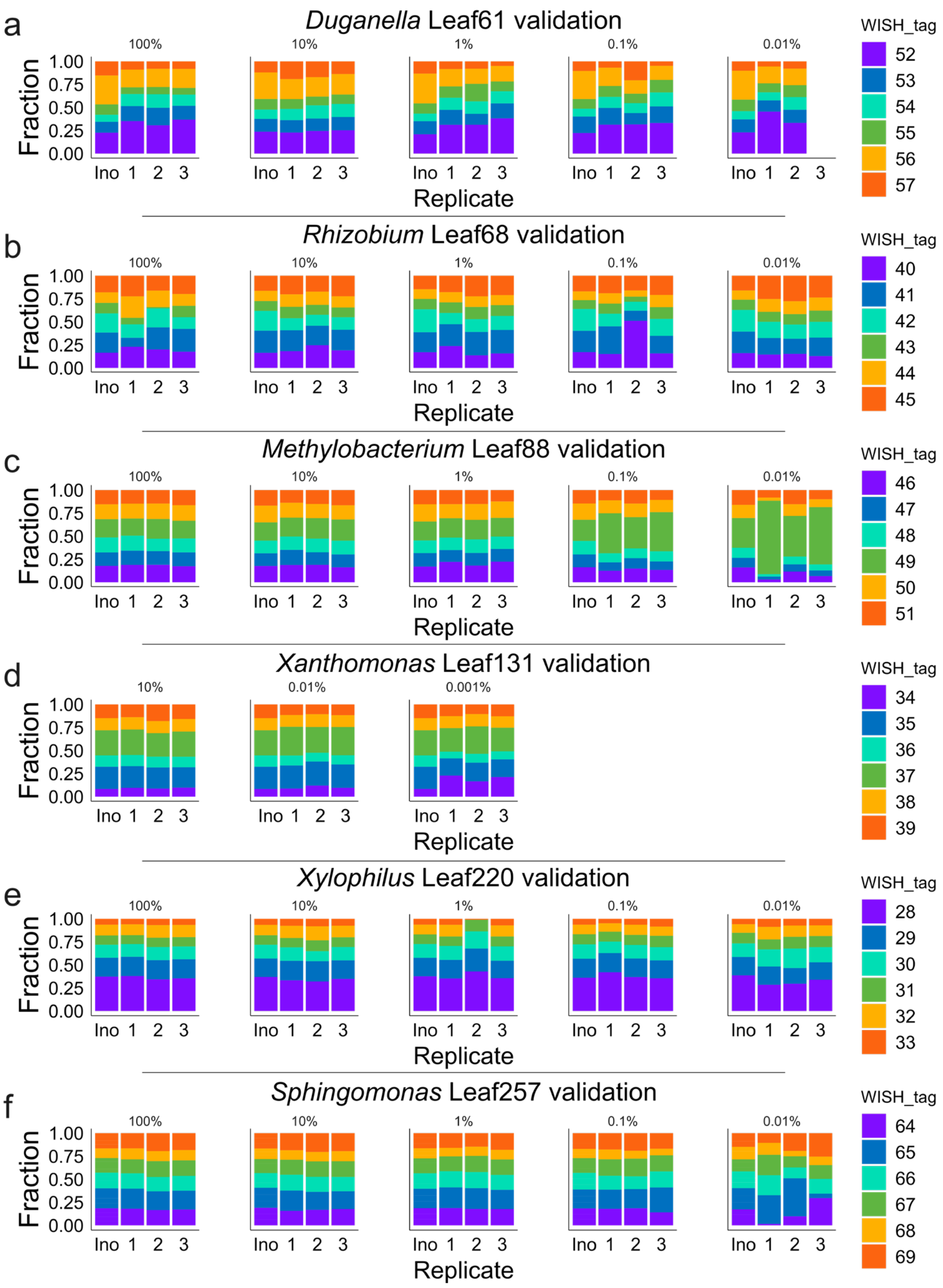

Supplementary Figure 5. *In vitro* validation of six *At*-LSPHERE strains to detect potential fitness effects due to tagging. Each strain harbors six unique WISH-tags that are indicated by color. Colors repeat across panels but refer to the WISH-tags indicated in the panel specific legend. The x-axis indicates the fraction each barcode contributes to the total barcoded population. The y-axis first shows the relevant inoculum, then three replicate cultures sampled after growth. Each panel consists of multiple plots, with an increasing amount of WT strain being added from left to right, the percentage above each plot as indicating the share of the barcoded population. a) *Duganella* Leaf61. b) *Rhizobium* Leaf68 c) *Methylobacterium* Leaf88 d) *Xanthomonas* Leaf131: The setup for the validation experiment was altered to accommodate additional controls that are not shown here. e) *Xylophilus* Leaf220 f) *Sphingomonas* Leaf257.

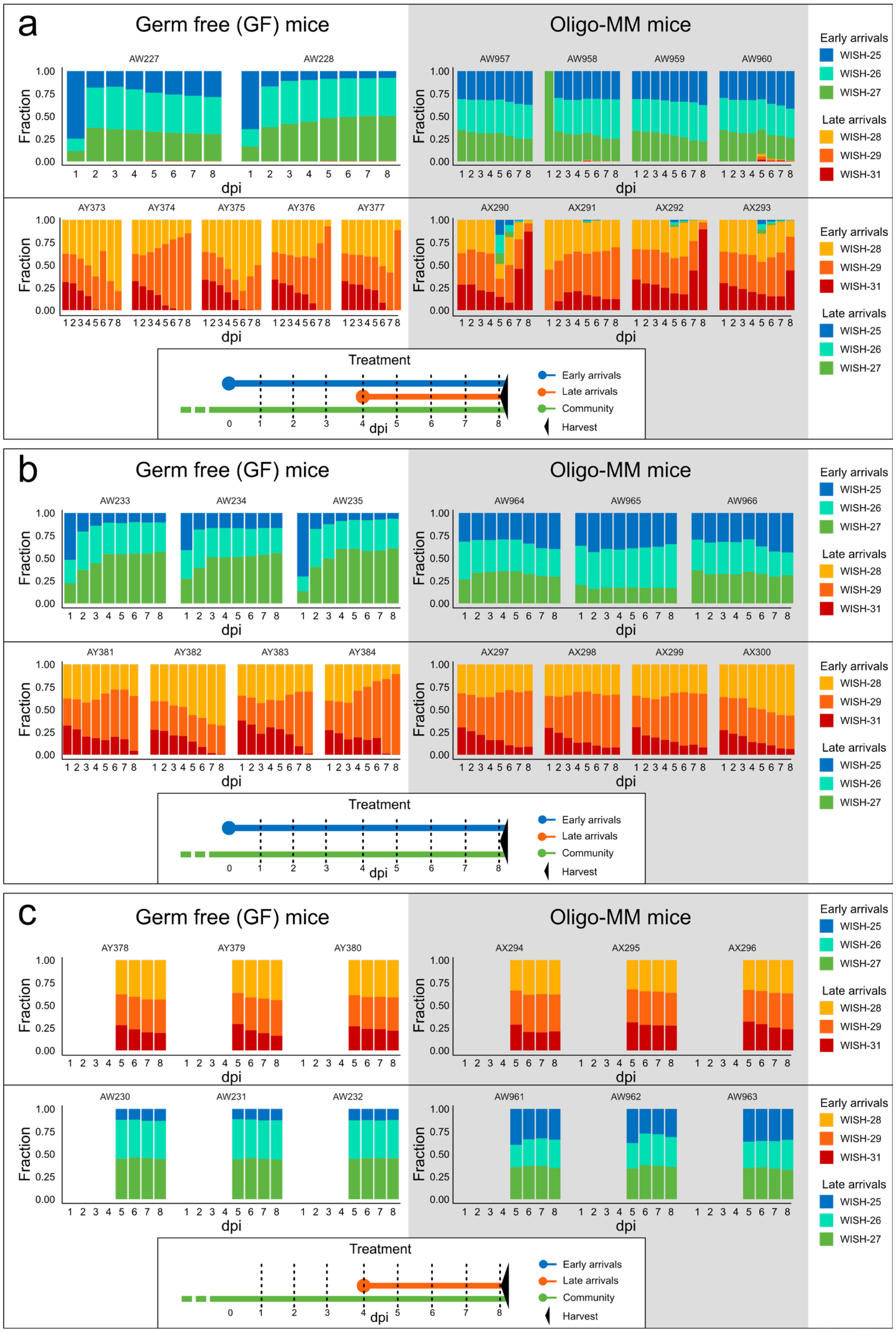

Supplementary Figure 6. **Treatment-dependent variation across individual mice**. Fractional-bar charts displaying the share of each WISH-tag in the total *S.* Tm population over the duration of the experiment with each subplot representing data from one mouse. The plots on the left represent the data obtained from germ-free (GF) mice, while the right half with the grey background, was generated from OligoMM^{12}-mice. The legend below the plots indicates the treatments, which were different between the mice shown in panels a), b), and c).

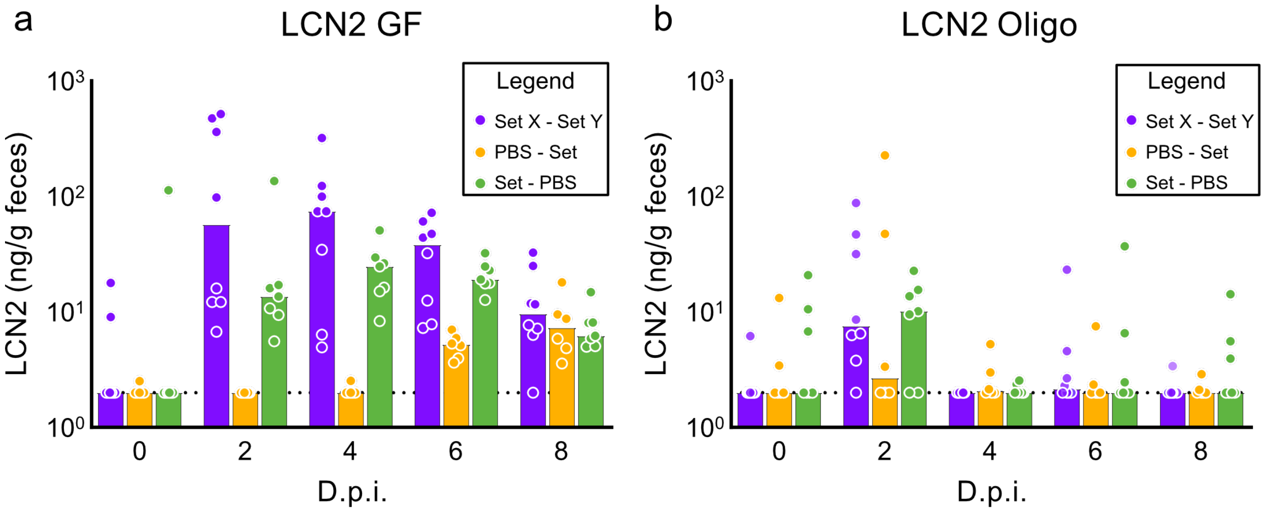

Supplementary Figure 7. Lipocalin-2 (LCN2) values as proxy for inflammation in the mouse gut. The x-axis indicates the day after inoculation the measurement was taken. The y-axis indicates the level of LCN2 measured in the feces. Each point represents a measurement from a different mouse. The height of the bars corresponds to the mean value. The dotted line indicates the detection limit. a) The LCN2 levels for the germ-free mice (for SetX-SetY, n = 8. For PBS-Set, n = 6 and for Set-PBS, n =7). b) The LCN2 levels for OligoMM^{12} mice (for SetX-SetY, n = 8. For PBS-Set, n = 6 and for Set-PBS, n =7).

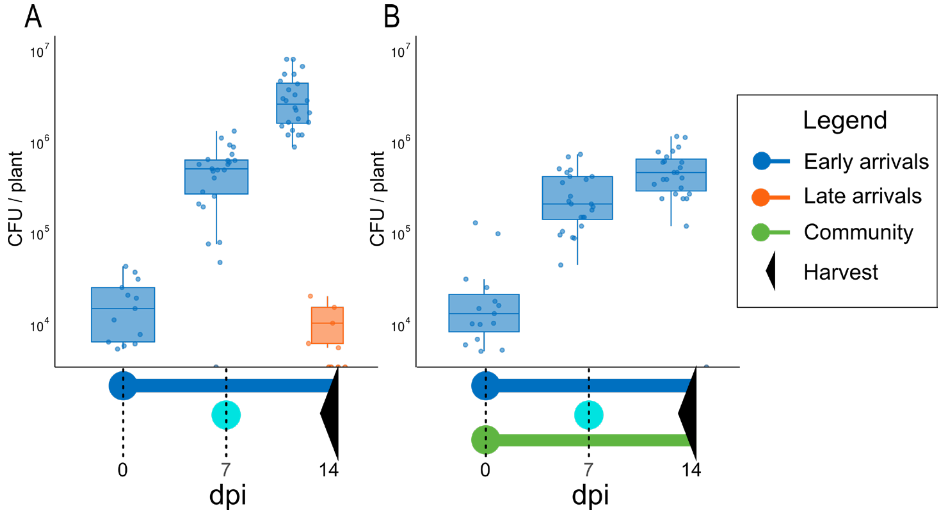

Supplementary Figure 8. Colonization levels of *Sphingomonas* in the phyllosphere in the presence and absence of a synthetic community. Colonization of *Sphingomonas* Leaf257 in mono association and upon co-colonization of a 15-strain microbiota community[11]. The plants were only inoculated with the early arrivals and then treated with a mock inoculation at 7 dpi to assess the removal of established bacteria by the process of inoculation. The x-axis shows the time and the y-axis the CFU per plant. The scheme below the plot illustrates the treatment (n=24 for each timepoint, the center line represents the median, with the upper and lower edges of the box representing the 25 and 75 percentile respectively. The whiskers extend at most 1.5 times the interquartile range). a) Data from plants solely colonized by *Sphingomonas* Leaf257. At 14 dpi, a low numbers of tags were detected that corresponded to the barcodes used for late arrivals. In five samples they reach ≤ 1% of the population, although they were not added to the plant, which might be due to the elevated level of noise in the sequencing run, see main text. This is the only condition where the noise surpasses the detection limit. b) Data from the plants that were inoculated with the 15-strain community.

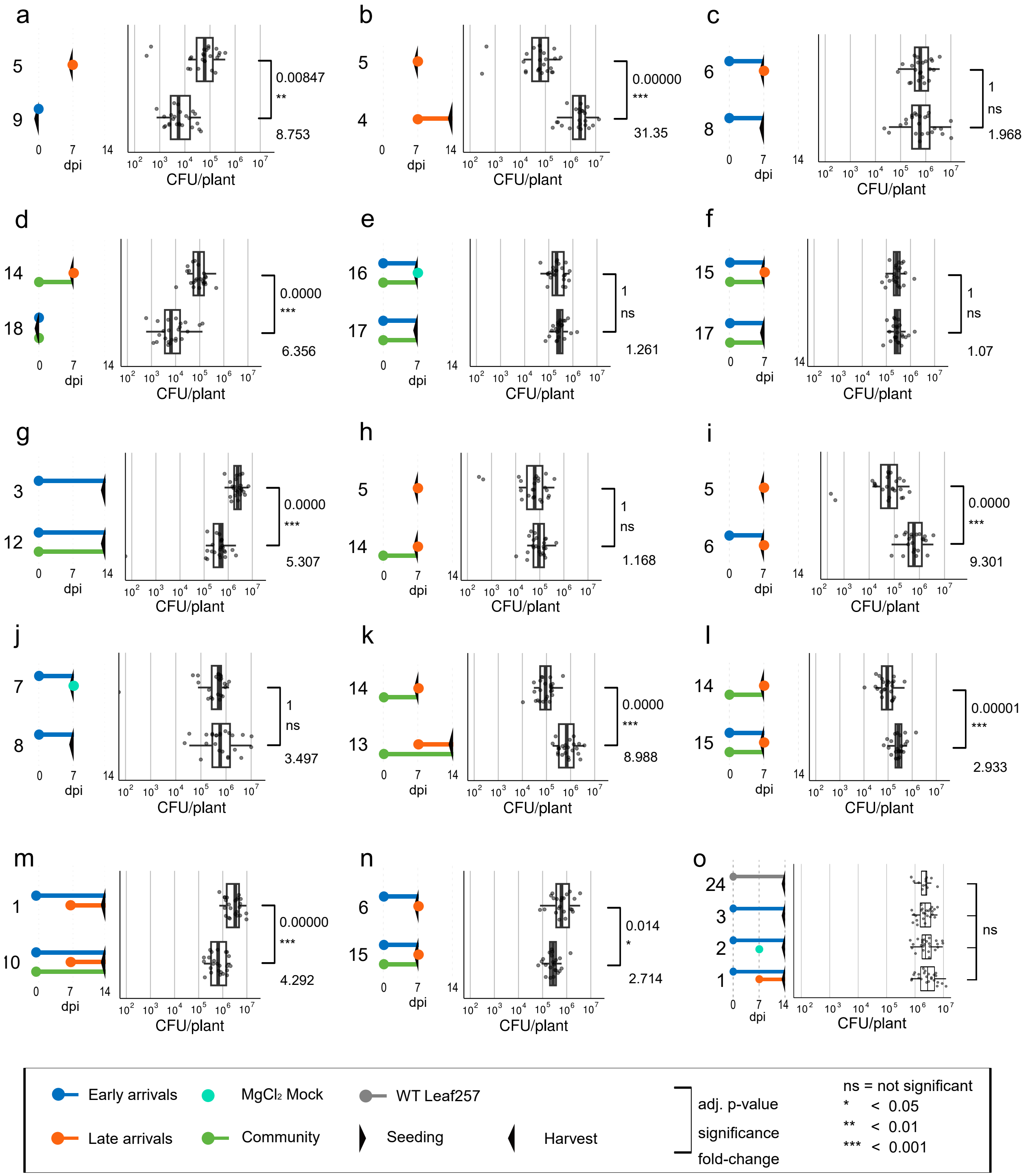

Supplementary Figure 9. Control conditions for experiments on early and late arrival of *Sphingomonas* Leaf257. The data are indicated per plant and are taken from the experiment shown in Figure 4. The x-axis shows the CFU recovered per plant, while the y-axis separates the two conditions that are being compared. A list of all conditions can be found in Extended Data Figure 2b. The center line represents the median, with the upper and lower edges of the box representing the 25 and 75 percentiles respectively. The whiskers extend at most 1.5 times the interquartile range. Various statistical models were assessed using the Akaike information criterion to analyze the data. The most appropriate models were selected for comparisons. Equal variance was examined using the Bartlett test, while normal distribution was confirmed using the Shapiro test. After meeting these criteria, the Welch-pairwise test was employed, and the resulting p-values underwent correction using the Bonferroni method. a) Comparison of the retention of bacteria to the plant for inoculations at 0 and 7 dpi (n = 24). b) Test for population increase from inoculation at 7 dpi to harvest at

14 dpi in the presence of the 15-strain community (treatment 4, n=23 and for treatment 5, n=24). c) Comparison of population size at 7 dpi between plants inoculated at 0 dpi (n= 24) and plants inoculated at both 0 and 7 dpi (n=22). d) Comparison of the retention of Leaf257 in presence of the 15-strain community after inoculation between plants inoculated at 0 dpi or at 7 dpi (n=24 for both). e) Controlling for wash-off of bacteria due to the inoculation at 7 dpi on plants inoculated with the community and Leaf257 at 0 dpi. Comparison of CFU recovered from plants with and without mock inoculation (n=24 for both). f) Comparison of the population of Leaf257 at 7 dpi on plants inoculated at 0 dpi only (n=24) with plants inoculated at 0 dpi and 7 dpi (n=23) in presence of the 15-strain community. g) Contrasting the final population at 14 dpi of Leaf257 inoculated at 0 dpi between plants with and without the 15-strain community (n=24 for both). h) Comparison of the retention of Leaf257 inoculated at 7 dpi on plants with and without established 15-strain community (n=24 for both). i) Comparison of the Leaf257 population at 7 dpi, from plants inoculated at 0 dpi (n=22) and 7 dpi to plants just inoculated at 7 dpi (n=24). j) Controlling for wash-off of bacteria by inoculation at 7 dpi on plants inoculated with Leaf257 at 0 dpi. Comparison of CFU recovered from plants with and without mock inoculation (n=24 for both). k) Visualization of the population growth of Leaf257 inoculated at 7 dpi from 7 dpi to 14 dpi in presence of the 15-strain community (n=24 for both). l) Comparison of the Leaf257 population on plants with the 15-strain community, between plants inoculated at 0 dpi only (n=24) or at 0 and 7 dpi (n=23). m) Visualization of the impact of the 15-strain community on the population of Leaf257. The plants were harvested at 14 dpi and inoculated at 0 and 7 dpi (n=24 for both). n) Visualization of the impact of the 15-strain community on the population of Leaf257. The plants were harvested at 7 dpi and inoculated at 0 dpi (n=22 for the plants without 15-strain community, and n=23 for the plants with 15-strain community). o) Comparison of the relative fitness of barcoded *Sphingomonas* Leaf257 with wild type (WT) *in planta* (n=12 for WT, and n=24 for the other treatments).

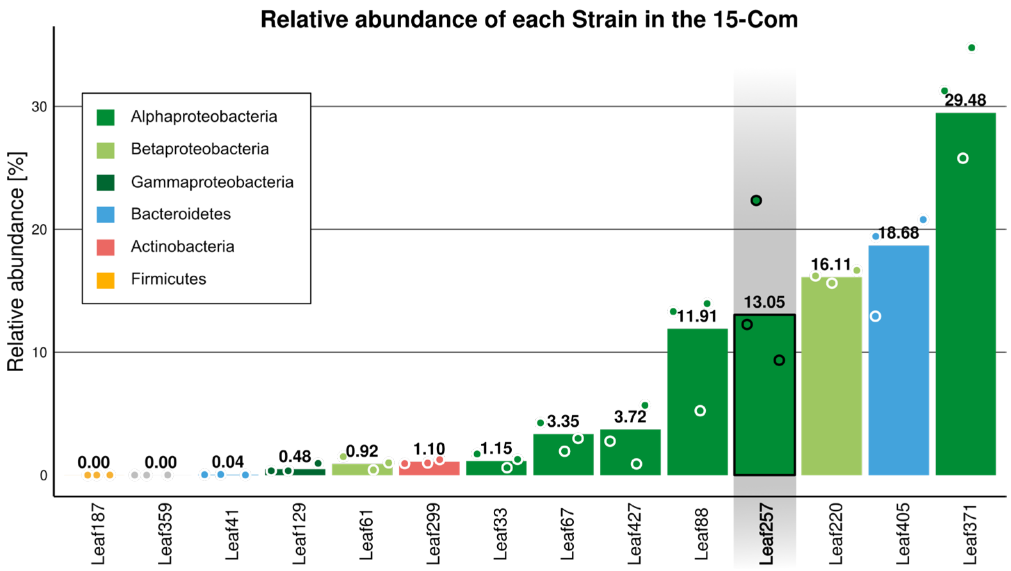

Supplementary Figure 10. Proportion of each strain in the 15-strain community in the phyllosphere, using data from Schäfer et al., 2022 (ref.[11]). *Sphingomonas* Leaf257 is highlighted. The x-axis lists the strains in increasing abundance, and the y-axis indicates the relative abundance of each strain in the total population. The number above the bar indicates the exact percentage (n=3, the top of the bar represents the mean of the replicates).

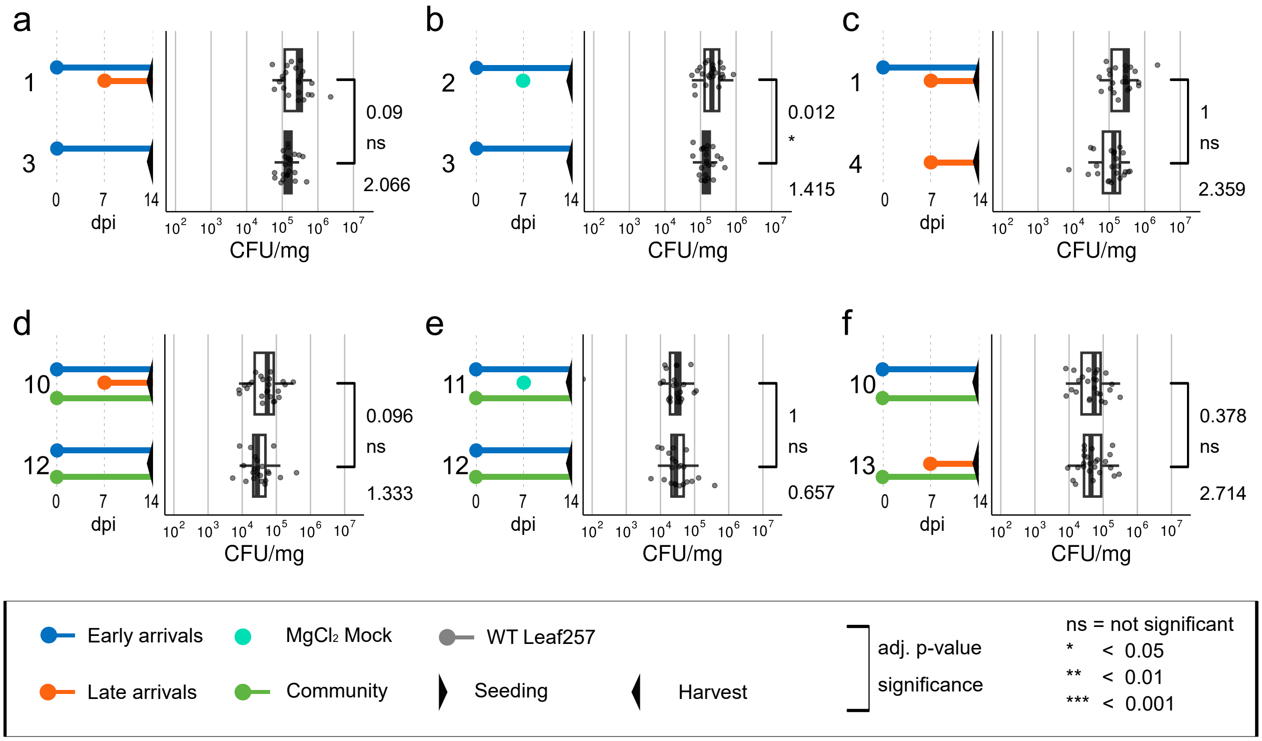

Supplementary Figure 11. Control conditions for experiment on early and late arrival of *Sphingomonas* Leaf257 per mg of plant weight. Comparison of treatments to account for experimental and external factors during the plant experiments shown in Figure 4. CFU normalized by plant weight for comparisons between controls. The x-axis shows the weight normalized CFU and the y-axis the different plant treatments. The scheme on the left of each plot indicates the treatments and the time point of application. A list of all conditions can be found in Extended Data Figure 2b. (the center line represents the median, with the upper and lower edges of the box representing the 25 and 75 percentiles respectively. The whiskers extend at most 1.5 times the interquartile range). a) Comparison of the colonization of Leaf257 between plants inoculated only at 0 dpi (n=23) versus plants inoculated at both 0 and 7 dpi (n=24 for both). b) Visualization of the impact of mock inoculation at 7 dpi on the final colonization of leaf 257 at 14 dpi (n=24 for both). c) Comparison of the final colonization between plants only inoculated 7 dpi (n=23) and those inoculated at 0 and 7 dpi (n=24). d) Comparison of the colonization of Leaf257 in presence of the 15-strain community, between plants inoculated only at 0 dpi versus plants inoculated at both 0 and 7 dpi (n=24 for both). e) Visualization of the impact of mock inoculation on the Leaf257 population in presence of the 15-strain community at 7 dpi on the final colonization of leaf 257 at 14 dpi (n=24 for both). f) Comparison of the final colonization of Leaf257 at 14 dpi, if the plant was only inoculated 7 dpi compared to an inoculation at 0 and 7 dpi in the presence of the community (n=24 for both).

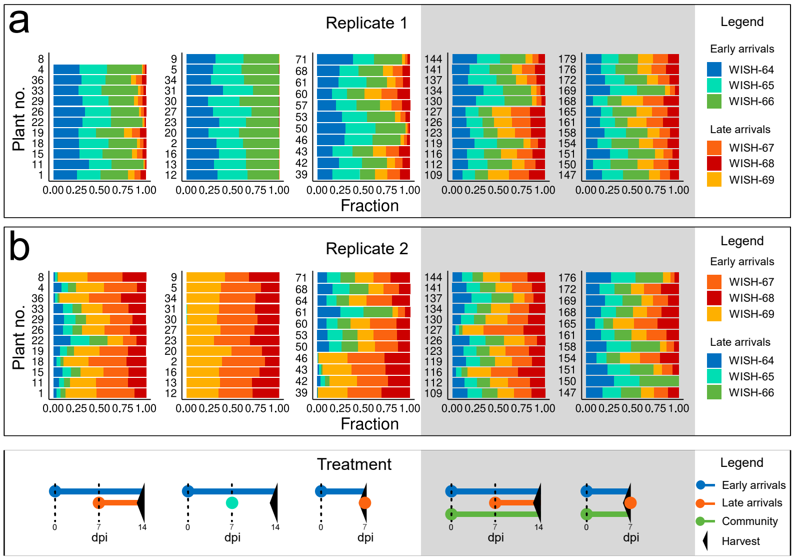

Supplementary Figure 12. Treatment-dependent variation across individual plants for key conditions shown in Figure 4. Fractional bar charts displaying the share of each WISH-tag in the total *Sphingomonas* Leaf257 population over the duration of the experiment. Each subplot represents one plant. The plots on the left represent the data of axenic plants, while the right half (grey background) is from the 15-strain community[11]. The figure legend below the plots indicates the treatments, which were different between the plants shown in the panels.

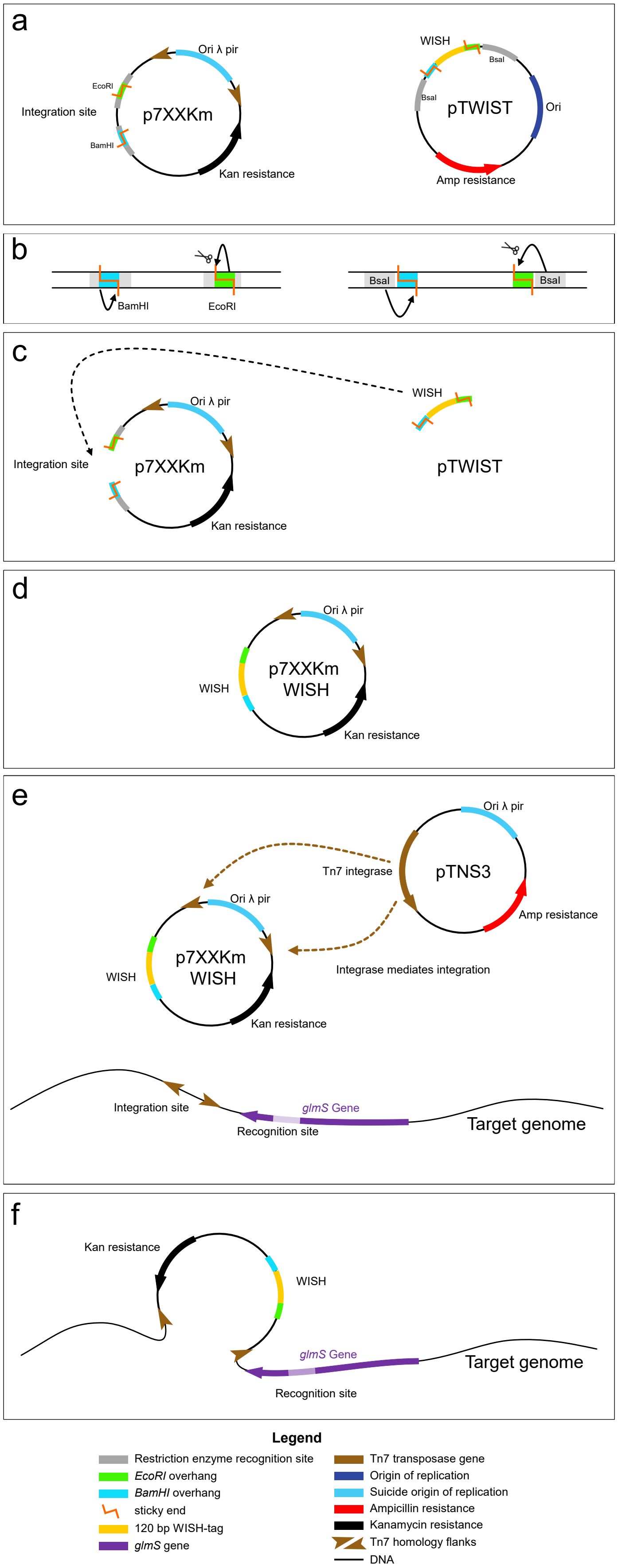
a
Ori λ pir
EcoRI
Integration site
p7XXKm
BamHI
Kan resistance
WISH
BsaI
BsaI
pTWIST
Ori
Amp resistance
b
BamHI
EcoRI
BsaI
BsaI
c
WISH
Ori λ pir
Integration site
p7XXKm
pTWIST
Kan resistance
d
Ori λ pir
p7XXKm
WISH
WISH
Kan resistance
e
Ori λ pir
Tn7 integrase
pTNS3
Amp resistance
Ori λ pir
p7XXKm
WISH
WISH
Integrase mediates integration
Kan resistance
Integration site
glmS Gene
Recognition site
Target genome
f
Kan resistance
WISH
glmS Gene
Target genome
Recognition site
Legend
Restriction enzyme recognition site
EcoRI overhang
BamHI overhang
sticky end
120 bp WISH-tag
glmS gene
Tn7 transposase gene
Origin of replication
Suicide origin of replication
Ampicillin resistance
Kanamycin resistance
Tn7 homology flanks
DNA

Supplementary Figure 13. Illustration of the cloning procedure for the integration of WISH-tags into *At*-LSPHERE strains, using p7XXKm as an example. a) Schematic depiction of the p7XXKm integration vector plasmid and the pTWIST high copy number plasmid carrying the WISH-tags. b) Close- up of the used restriction sites of the two plasmids. c) On the pre-existing p7XXKm plasmid, EcoRI and BamHI restriction sites were used, necessitating a separate double digestion step. The pTWIST plasmids were designed to contain two BsaI restriction sites. These were created so that digestion with BsaI results in EcoRI and BamHI compatible overhangs for GoldenGate cloning. This enables a one pot reaction in which the pre-digested p7XXKm backbone was added, combining ligation and digestion in one highly efficient step. d) Completed integration vector carrying a WISH-tag. e) To incorporate the WISH-tag into the target genome, the integration vector and a helper plasmid, here pTNS3, have to be transformed into the target strain simultaneously. The helper plasmid pTNS3 expresses the Tn7 integrase facilitating the integration of the WISH-tag and a selectable marker via the Tn7 homology flanks. f) Due to the suicide vectors on both p7XXKm and pTNS3, the plasmids are lost after growth of the modified strains.

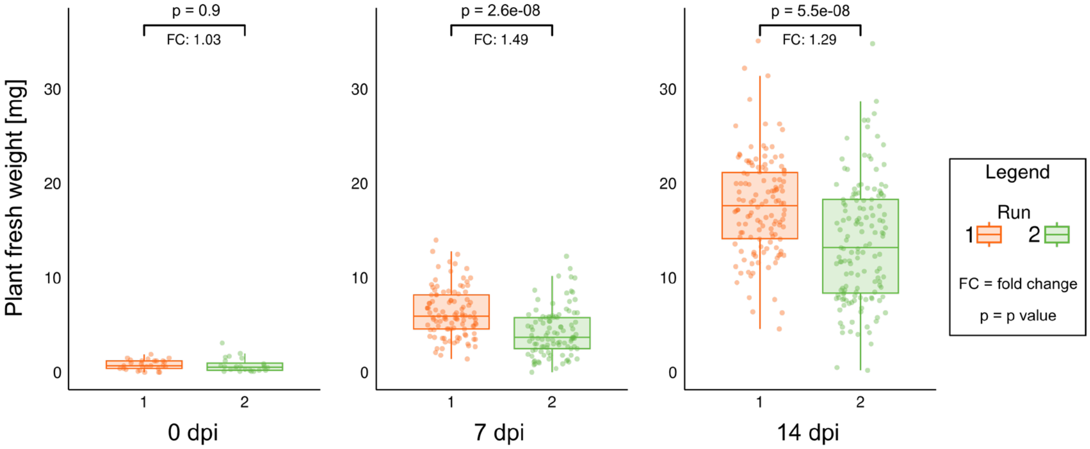

Supplementary Figure 14. Comparison of plant weights between the first and second replicate experiments (Run 1 and 2). The x-axis indicates the run and the y-axis the plant fresh weight in mg. The plants of the second replicate were significantly smaller (at day 7, a difference of 1.5 was observed, on day 14 a difference of 1.29). The fold change was calculated by dividing the larger mean by the smaller mean (the center line represents the median, with the upper and lower edges of the box representing the 25 and 75 percentiles respectively. The whiskers extend at most 1.5 times the interquartile range. n=36 for Run1 and Run2 at 0 dpi. At 7 dpi, n=107 for Run1 and n=106 for Run2. At 14 dpi, n=132 for Run1 and n=143 for Run2).
